# Inferred Rate of Default as a Credit Risk Indicator in the Bulgarian Bank System †

**DOI:** 10.3390/e25121608

**Published:** 2023-11-30

**Authors:** Vilislav Boutchaktchiev

**Affiliations:** 1 Faculty of Applied Informatics and Statistics, University of National and World Economy, 1700 Sofia, Bulgaria; vboutcha@unwe.bg; 2 Institute of Mathematics and Informatics, Bulgarian Academy of Sciences, 1113 Sofia, Bulgaria

**Keywords:** probability of default, ARMA estimation, Vasicek model, random walk, IFRS9, expected credit loss, 62M05, 62N02, 91B70, G21, M41

## Abstract

The *inferred rate of default (IRD)* was first introduced as an indicator of default risk computable from information publicly reported by the Bulgarian National Bank. We have provided a more detailed justification for the suggested methodology for forecasting the IRD on the bank-group- and bank-system-level based on macroeconomic factors. Furthermore, we supply additional empirical evidence in the time-series analysis. Additionally, we demonstrate that IRD provides a new perspective for comparing credit risk across bank groups. The estimation methods and model assumptions agree with current Bulgarian regulations and the IFRS 9 accounting standard. The suggested models could be used by practitioners in monthly forecasting the point-in-time probability of default in the context of accounting reporting and in monitoring and managing credit risk.

## 1. Introduction

The *inferred rate of default*, IRD, for Bulgarian banks was introduced in [1] and was motivated by the need for models for forecasting the probability of default. It is calculated entirely from publicly available data and approximates the actual rate of default.

Recall that the *probability of default*, PD, of a bank loan is the probability of reaching a state where the borrower is unlikely to repay the amount due over a certain time horizon.

The *rate of default* $RD_{t}$, furthermore, is the observed data point for the probability of default at the end of period *t*. It is calculated by collecting a portfolio of credits with a set risk profile and tracing the amounts transitioning between states over a given period:

(1) $$RD_{t}=\frac{N_{t}}{P_{t-1}}.$$

Here, $P_{t-1}$ is the amount of all performing loans at the beginning of the period *t*, and $N_{t}$ is the amount of those performing at the beginning but defaulting at the end of period *t*. In this study, we consider exclusively rates of default over 12 months. (This is the horizon methodologically most useful in estimating the expected credit loss.)

Following the IFRS 9 accounting standard, the regulator has postulated a set of rules by which the banks may determine at any time which borrower is unlikely to repay. Among those, the most definitive and commonly applied is that a contract with more than 90 days of delinquency is considered in default.

Although banks are constantly monitoring and periodically reporting rates of default to the regulator, these reports are considered confidential. Public disclosure of the quality of their exposures is given by some banks in their annual financial reports. Nonetheless, the regulator periodically publishes carefully selected data on the banks’ assets’ quality, not about individual banks, but only on a systemic level.

With the introduction of the IFRS 9 accounting standard, Bulgarian banks were required to employ models to produce economically justified forecasts of the probabilities of default for the estimation of their expected credit loss (cf., e.g., [2]). The development of such forecasting models for point-in-time PD is a subtle task because the results should not be perceived as overly optimistic or excessively conservative, since both could bring additional accounting risks (cf., e.g., [3]).

For this reason, the banks are encouraged to build their own forecasting policies using available private and public data. The banks’ own data on rates of default, however, are often found in need of corrections due to errors, omissions, or corporate events which contaminate the data.

In the context of ECL estimation, there has been a large volume of literature on PD forecasting, including normative documents (cf., e.g., [2]); studies of credit risk (cf., e.g., [4,5,6,7,8]); economic factors determining market performance (e.g., [9]); general forecasting techniques (e.g., [10,11,12,13,14,15]); and the relationship between point-in-time and through-the-cycle PD is developed in detail in, e.g., [16].

For Bulgarian banks, previously, in [17], PD forecasting models were tested using privately acquired data for the actual rates of default. Here, an ARMA model reveals both the strong dependence from macroeconomic factors, as well as characterizing the autoregressive nature of the RD series.

The IRD is defined on a systemic level for large groups of banks in [1], where models for its forecasting were built particularly for the portfolios of Group 2 banks, among which were also the institutions whose data were used in [17]. In [1], the similarity of the ARMA forecasting models that govern the RD and IRD was pointed out as evidence that both series measure credit risk in a similar way. This argument is recalled in Section 4.4 for comparison. Also, in [1], the asymptotic single risk factor (ASRF) model is defined and Proposition 1 is formulated without proof.

In the current study, we extend the IRD to three groups of banks. The portfolio selection is detailed in Section 2 where we recall the definition of IRD and comment on the descriptive statistics of the data used in the empirical analysis. In Section 3, we revisit the asymptotic single risk factor model for the probability of inferred impairment, providing a fuller and more detailed justification of the methodology. The main result is Proposition 1, which gives validity to the subsequent empirical estimation. The parameters of models of this type are estimated in Section 5.

In Section 4 and Section 5, we develop forecasting models empirically through time-series analysis. Section 4 demonstrates models based on ARMA regressions, designed especially to demonstrate comparison with the findings from [1,17].

While in [1] the primary focus was on proving the similarity between the IRD and RD as measures of credit risk, here, in Section 6, we introduce a cross-sectional analysis of credit risk between different types of clients and institutions.

Further steps in the IRD study would involve the application of the techniques of event study and principal component analysis (similarly to, e.g., [13,18]) to analyze the effect of various system-wide events and regulatory changes.

More generally, the idea of analyzing the risk of failure using revealed or publicly disclosed data has applications that are not limited to banking (cf., e.g., [19,20]) but can be extended to engineering fields (e.g., speech, [21]), where loss estimation is needed. Further development of our methodology in this direction will be a subject of future study.

## 2. Inferred Rate of Default

To define the inferred rate of default we take advantage of the quarterly reports on the credit quality of banks. These reports have been issued under different titles through the years: *Information about credit quality and impairments* till the end of 2014, and since 2015 as *Information on nonperforming loans and impairments*. Despite minor variances in the classification, we were able to identify the necessary quantities during the entire period 2007–2022. In 2019, with the coming of the COVID-19 pandemic, the Bulgarian government introduced a series of measures that gave the banks the option to postpone the recognition of failing loans as “defaulted”. This has had the effect of “smoothing” the rates of default and inferred rates of default during that period. To avoid contamination, we have omitted from consideration all data after 31 December 2019.

These reports provide a classification of the bank exposures based on the *type of borrowers*, e.g., banks, non-financial corporations, households, etc. Within each portfolio, the report identifies the amounts that, at the end of the reporting period, *t*, fall into the following four categories:

- **Regular-performing loans.** These are loans that continue without any violation of the contract. We call this segment S1 and denote the total amount of the loans in it by $P_{t}$
- **Loans in breach of contract.** These are further sub-classified into three groups based on the length of the delinquency period:
  − Loans past-due no more than 90 days, denoted by S2 with total $P_{t}^{\prime }$. These loans were considered *under review* for most of the period of available data using the previous accounting regulations, and currently most of them fall into Stage 2 of impairment under IFRS 9.
  − Loans past-due more than 90 but less than 181 days, *nonperforming*, denoted by S3 with total $N_{t}$
  − Loans past-due more than 180 days, *lost*, denoted by S4 with a total $N_{t}^{0}$

We construct the *inferred rate of default* as follows:

(2) $$IRD_{t}=\frac{N_{t}+\max\left\{0,N_{t}^{0}-N_{t-1}^{0}\right\}}{P_{t-1}+P_{t-1}^{\prime }}.$$

We concentrate on two types of borrowers only:

1. **Non-financial corporations.** These are loans extended to corporations, excluding any state and local governments and their subsidiaries, as well as banks and other financial institutions
2. **Households.** These are retail credits, including all consumer loans and mortgages.

The data are provided for aggregated portfolios for *bank groups*. The Bulgarian National Bank (BNB) classifies the banks in the country (ever since 2007) into three categories in the following manner: Group 1 consists of the five largest banks, Group 3 are branches of foreign banks, and Group 2 are all the rest.

Group 3 contains four to six branches of large foreign banks, most of which take a specific niche in the Bulgarian market. This portfolio is excluded from this analysis for three reasons. First, these institutions are not under the same domestic regulatory process as the rest. Second, there are some inconsistencies in the BNB reports in this group. Finally, because of the small size of the Group 3 portfolio, corporate events like, e.g., a merger between two banks or the addition of a new bank to the group, causes significant disruption of the time series. However, the data from these banks are still part of the reports on the total bank system.

Table 1 shows summary statistics of the inferred rates of default for the two segments, *retail* and *corporate* in three systemic portfolios: the whole bank system, Group 1, and Group 2. The initial overview of the IRD confirms the inherent characteristics of the bank groups.

- The data for Group 1 has a lower mean than Group 2 in both the corporate and retail portfolios. This indicates that Group 1 banks have the tendency to attract clients with better credit quality.
- In the corporate portfolio, the IRD of Group 1 banks shows a higher variation than Group 2. Considering that the corporate clients in a bank are often carefully and individually managed, this might be evidence that Group 1 banks are in a position to pursue a more aggressive policy in new client taking.
- The variance in the retail portfolio is lower for Group 1. Retail portfolios are usually managed collectively, through general policy and automatic decisions, hence, lower variance is evidence of more effective management.

The Group 1 banks are the leaders in the market, while Group 2 consists of 15–20 small institutions that are market followers, some of which are built to satisfy a specific need in the Bulgarian economy. Section 6 is dedicated to a comparative analysis of the two groups of banks and the two types of clients based on the IRD.

### 2.1. Economic Interpretation of the Inferred Rate of Default

Similarly to the usual RD, the IRD can be studied as the historic relative frequency of a probabilistic distribution.

**Definition** **1.** *The event of a loan transitioning from states S1 or S2 to S3 or S4 during year t is referred to as inferred impairment and its expected relative frequency using the information set $S_{t-1}$, available 1 year back*

$$PII_{t}=E\left[IRD_{t}|S_{t-1}\right]$$

*is called the probability of inferred impairment*.

As the rate of default measures the relative frequency of loans transitioning from the regular state to default, the *inferred rate of default* is designed to estimate the probability of transitioning from regular to inferred impairment.

**Remark** **1.** *The event of inferred impairment is costly for the bank and, hence, for the contract holder as well.*

We list here some of the key differences between the RD and IRD.

1. While *default* is the event of the bank concluding that the debtor will be incapable of repaying its debt, the event of *Inferred impairment* signifies that the bank has made this same conclusion in the past 3–6 months. This delay may cause the IRD to be larger than the actual RD.
2. In constructing the IRD, the loans past-due less than 90 days are considered as performing. Most of them, very likely, would have been considered as such in the computation of ECL, even with an elevated risk (i.e., classified in stage 2 of impairment, cf., [3]). Some of them, however, should have been regarded as defaulting, according to the individual bank’s policies. This eventuality, however rare, may cause the IRD to be smaller than the RD.
3. While it is, in most situations, fair to consider loans less than 6 months past due as fresh NPE for the given quarterly report, some of them will probably have been classified as defaulting by the banks even earlier. The IRD catches such cases with eventual delay.
4. We consider in the IRD the positive growth in stale NPE (past-due more than 180 days) as recent defaults. Our experience indicates that banks often manage the riskier loans in their portfolio in a way that allows them to record newly defaulting loans directly into this group. This is partly due to their policies being formulated during an earlier regulatory period which allowed loans to be considered as “exposure under review” up until the moment when they was declared “lost”. On the other hand, excluding these exposures from consideration would have made the IRD uncharacteristically small at this time. Perhaps, in the future, as regulation and supervision become more diligent, this component will have to be modified or even excluded from consideration.

### 2.2. Macroeconomic Factors

The explanatory power of macroeconomic conditions as predictors of debtor behavior is well documented. It is argued in, e.g., [17] that the probability of repayment of a corporate loan is negatively correlated to the growth in the national GDP, and similarly, in the retail portfolio, the PD depends on the unemployment rate observed in the labor market. For this reason, we limit our study to these two macroeconomic indicators.

In the building of forecasting models for the IRD, we use the reports of the National Statistical Institute (NSI) of Bulgaria for the gross domestic product (GDP) and the unemployment rate.

For the estimation of the ASRF model to work properly, we need the assumption that the period of analysis, 2007–2019, covers a full economic cycle. This requirement is very likely satisfied since the period spans through one severe worldwide financial crisis (2008), as well as a period of steady growth in the national economy (2014–2018).

#### 2.2.1. Gross Domestic Product

We use real GDP quarterly reports to compute the one-year growth rate of annual GDP at the end of each quarter.

$$G\left(t\right)=\frac{RGDP[t-3,t]}{RDGP[t-4,t-7]}-1.$$

Here, RGDP[t−3,t] denotes the cumulative real GDP at the average 2015 prices as reported in the four quarters t−3,…,t.

#### 2.2.2. Unemployment Rate

The unemployment rate is reported by NSI at the end of each quarter and it is measured as the number of unemployed persons as a proportion of the labor force. The variable used for this analysis is the annual growth or the rate of default:

$$U\left(t\right)=\frac{UR[t]}{UR[t-4]}-1.$$

Here, UR[t] denotes the unemployment rate measured at the end of quarter *t*. The NSI also reports an *annualized* (average) unemployment rate, which turns out to be less suitable for the ARMA regressions.

## 3. A Modified Vasicek Model

In this section, we build an asymptotic single risk factor (ASRF) model for the probability of inferred impairment, similar to the one used for the probability of default. For this purpose, we will distinguish two different measures of the 12-month PII: the *through-the-cycle PII* (TTC-PII), which is the expected PII over a total economic cycle, and the *point-in-time PII* (PIT-PII), which is the actual value at time *t*.

Vasicek’s ASRF model (cf., e.g., [22]) applies a geometric Brownian motion equation to study a firm’s asset value:

(3) $$dA_{i}=\mu_{i}A_{i}dt+\sigma_{i}A_{i}dx_{i},$$

where $A_{i}$ is the individual borrower’s asset value, $\mu_{i}$ and $\sigma_{i}$ are the drift rate and volatility of that value, and $x_{i}$ is a Wiener process. Equation (3) has an analytic solution under Itô’s interpretation, hence, the value $A_{i}$ at the loan’s maturity time *T* is represented as

$$\ln A_{i}\left(T\right)=\ln A_{i}\left(0\right)+\mu_{i}T-\frac{1}{2}\sigma_{i}^{2}T+\sigma_{i}\sqrt{T}X_{i},$$

where $X_{i}$ has a standard normal distribution. Assuming that the total debt of the borrower is $B_{i}$, the probability of default (cf., [23]) is

$$PD_{i}=P\left[A_{i}\left(T\right)<B_{i}\right]=P\left[X_{i}<d_{i}\right]=\Phi \left(d_{i}\right).$$

Here, Φ is the cumulative density function (CDF) of the standard normal distribution and

$$d_{i}=\frac{\ln B_{i}-\ln A_{i}\left(0\right)-\mu_{i}T+\frac{\sigma_{i}^{2}T_{i}}{2}}{\sigma_{i}\sqrt{T_{i}}}$$

is the default threshold of the borrower.

As noted earlier, in Remark 1, although the transition of a given exposure to the state of inferred impairment does not necessarily lead to the total loss of the firm, it is costly for the borrower. Hence, we may assume that it will be allowed to happen only in cases where the asset’s value has dropped below a certain level, ${\tilde{B}}_{i}$. Therefore,

(4) $$PII_{i}=P\left[A_{i}\left(T\right)<{\tilde{B}}_{i}\right]=P\left[X_{i}<D_{i}\right]=\Phi \left(D_{i}\right).$$

We will call

(5) $$D_{i}=\frac{\ln{\tilde{B}}_{i}-\ln A_{i}\left(0\right)-\mu_{i}T+\frac{\sigma_{i}^{2}T_{i}}{2}}{\sigma_{i}\sqrt{T_{i}}}$$

the *inferred impairment threshold* of the asset.

Furthermore, we make a standard (cf., [5,22]) assumption, that a bank portfolio is built of *n* assets with a similar risk profile, resulting in the constraint that the respective variables $X_{i}$ are jointly standard normal and of equal pairwise correlation ρ. We have

$$X_{i}=Z\sqrt{\rho }+Y_{i}\sqrt{1-\rho },$$

where $Z,Y_{1},\ldots Y_{n}$ are mutually independent standard normal variables. In this setup, *Z* is considered as a *common factor* which has an effect on the portfolio (e.g., the overall macroeconomic and business cycle over the interval [0,T]), $Y_{i}$ represents the borrowers’ respective *idiosyncratic risk*, and $\rho =\mathrm{corr}(Z,X_{i})$ is the *asset correlation*.

**Definition** **2.** *The point-in-time PII of an asset is the function*

$$p_{i}\left(z\right)=P\left[X_{i}<D_{i}|Z=z\right]=\Phi \left(\frac{D_{i}-z\sqrt{\rho }}{\sqrt{1-\rho }}\right),$$

*i.e., the probability of inferred impairment given the systemic factor has a specific value Z=z.*

**Definition** **3.** *The through-the-cycle PII of an asset is obtained as the marginal probability by averaging through all possible macroeconomic conditions (i.e., values of Z)*

$$p_{i}=E_{Z}\left[p_{i}\left(Z\right)\right]=\int_{-\infty }^{\infty }p_{i}\left(z\right)\phi \left(z\right)\;dz=\Phi \left(D_{i}\right).$$

Although originally developed for an individual asset, the Vasicek setup applies to the entire portfolio of loans, instead. In this case, for a portfolio

- the PIT-PII is given as
  (6) $$p\left(z\right)=\Phi \left(\frac{D-z\sqrt{\rho }}{\sqrt{1-\rho }}\right),$$
- the TTC-PII is computed as

(7) $$p=E_{Z}\left[p\left(Z\right)\right]=\Phi \left(D\right),$$

where *D* can be interpreted as the inferred impairment threshold of the portfolio’s “average client”, or the “central tendency client”.

The parameters *D* and ρ can be estimated using the observed data of default rates. This type of estimation is sometimes performed using a methodology involving Kaplan filters and Monte Carlo integration (cf., e.g., [24,25]; implementation of this technique is left for the future). Here, we suggest a simpler application of a method of moments that produces a satisfactory approximate result. In the following proposition, we summarize the conclusions made above:

**Proposition** **1.** *In the setup of the Vasicek model with the additional assumption that both the asset correlation (i.e., the parameter ρ ) and the factor Z remain invariant over a prolonged period, we denote by $\{IRD_{t}$: t∈A} the time series of observed inferred rates of default (i.e., the measurements of PIT-PI). Then, the sample $\{\Phi^{-1}\left(IRD_{t}\right)$: t∈A} is drawn out of a normal distribution with mean μ and variance $\sigma^{2}$:*

$$\mu =\frac{D}{\sqrt{1-\rho }},\;\sigma^{2}=\frac{\rho }{1-\rho }.$$

**Proof.**  The normal distribution of the PII follows from Equations (4) and (5). Furthermore, Equation (6) implies that

$$E\left[\Phi^{-1}\left(IRD_{t}\right)\right]=E_{Z}\left[\frac{D-z\sqrt{\rho }}{\sqrt{1-\rho }}\right]=\frac{D}{\sqrt{1-\rho }},$$

and

$$Var\left[\Phi^{-1}\left(IRD_{t}\right)\right]=Var\left[\frac{D-z\sqrt{\rho }}{\sqrt{1-\rho }}\right]=\frac{\rho }{1-\rho }.$$

□

## 4. ARMA Regressions

In this section, we develop forecasting models for the IRD using an ARMA time-series analysis. We justify the choice of macroeconomic factors and provide evidence of empirical similarity between the IRD and RD. This process is repeated independently for the portfolios of Group 1, and Group 2, as well as the whole bank system. In Section 6, we compare bank groups and portfolios.

### 4.1. Explanatory Variables

We test the dependence between the macroeconomic indicators and the IRD in a sequence of linear regressions, reported in Table 2.

The results shown are from an ordinary least squares (OLS) regression estimation of the following models:

(8) $$y_{t}=\alpha +\beta x_{t-\tau }+\epsilon_{t}$$

Here,

$$y_{t}=\ln\left(IRD_{t}\right)-\ln\left(IRD_{t-4}\right)$$

is always the annual logarithmic growth in the IRD, τ=0,1,…,4 is a time lag in quarters, and the explanatory variables are defined as follows:

- For the corporate portfolio:
  $$x_{t}=G\left(t\right)-G\left(t-4\right).$$
- For the retail portfolio:
  $$x_{t}=U\left(t\right)-U\left(t-4\right).$$

In the corporate portfolio, we observe a strong negative dependence on lagged changes in GDP. The results indicate a difference in the response of the two bank groups. For the IRD of Group 1 and the whole bank system portfolios, the four-quarter-old macroeconomic factor is the best choice in size, statistical significance, and explanatory power of the response. For Group 2, the IRD adjusts immediately to changes in the macroeconomic climate. (In practice, official data on GDP growth are available at least five quarters after the end of the period. Before that, the data market offers a variety of forecasts, which differ in accuracy. Some are independent and authoritative, while others show a political bias. Sufficiently accurate express estimates are usually available three months after the end of the period).

In all groups, the IRD of the retail portfolio shows an immediate positive response to changes in the dynamics of the unemployment rate.

The stationarity of all the time series, x(t) and y(t), is confirmed using the Dickey–Fuller test.

### 4.2. Corporate Portfolio

Using the Box–Jenkins method we identify the best-fitting models.

For Group 1, as well as the whole bank system, we have an MA(1) model:

(9) $$y_{t}=\beta x_{t-4}+\theta \epsilon_{t-1}+\epsilon_{t}$$

In Group 2, we have an ARMA(1, 1) model:

(10) $$y_{t}=\beta x_{t}+\rho y_{t-1}+\theta \epsilon_{t-1}+\epsilon_{t}$$

The results of the estimation are shown in Table 3.

### 4.3. Retail Portfolio

We apply the Box–Jenkins scheme to select an AR(4) model as best fitting:

(11) $$y_{t}=\beta x_{t-\tau }+\rho_{4}y_{t-4}+\epsilon_{t}$$

The results of the estimation are shown in Table 4.

For the portfolio in Group 2, we report the result of the model AR(1), which fits reasonably well and has a similar form to the one reported in [17]:

(12) $$y_{t}=\beta x_{t-\tau }+\rho_{1}y_{t-1}+\epsilon_{t}$$

### 4.4. Comparison with Rate of Default

The comparison with the forecasting models for the rate of default was a central topic of [1]. The models for the IRD for Group 2 bear a certain resemblance with those for RD developed in [17]:

- For the corporate portfolio:
  $$y_{t}=-6.96x_{t}+0.86y_{t-1}-0.99\epsilon_{t-1}+\epsilon_{t}.$$
- For the retail portfolio:
  $$y_{t}=0.86x_{t-1}+0.697y_{t-1}+\epsilon_{t}.$$

This similarity suggests that the IRD distribution can be used to build models for the replacement of missing and faulty data.

## 5. ASRF Model Estimation

For each of the retail and corporate portfolios, we denote with $\hat{\mu }$ and $\hat{\sigma }$, respectively, the sample mean and standard deviation of the series $\Phi^{-1}\left(IRD_{t}\right)$ of inferred rates of default. Based on Proposition 1, we then estimate

$$D=\frac{\hat{\mu }}{\sqrt{1+{\hat{\sigma }}^{2}}},\;\rho =\frac{{\hat{\sigma }}^{2}}{1+{\hat{\sigma }}^{2}}$$

and

$$Z_{t}=\frac{\hat{\mu }-\Phi^{-1}\left(IRD_{t}\right)}{\hat{\sigma }}.$$

Setting

$$y_{t}=Z_{t}-Z_{t-4},$$

we confirm that the forecasting models (9)–(11) are valid for all the portfolios. The results of the estimation are shown in Table 5 and Table 6.

**Remark** **2.** *A direct linear estimation of the IRD is impractical because the values belong in the interval (0, 1). In the original ARMA model, we applied a logarithmic transformation to remedy this. The ASRF model technically differs only in the use of the inverse normal CDF as a means to rescale the response variable. In a deeper sense, however, the difference is that the transformation of the Vasicek model is theoretically motivated, which allows the underlying assumptions of the estimation process to be clarified.*

The Vasicek model brings only a minor improvement in the accuracy of prediction. Table 7 shows the comparison in the standard error of estimation between the two techniques.

## 6. Cross-Sectional Analysis

**Figure 1:**
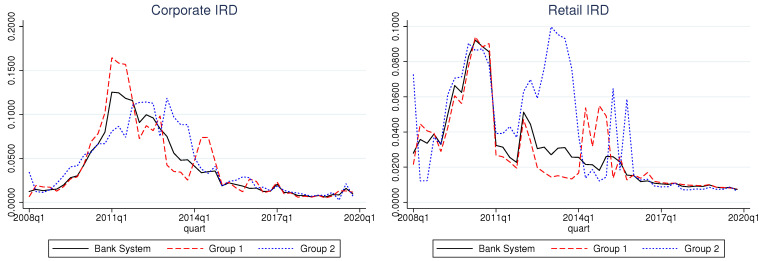
A comparison between inferred rates of default in Group 1 and Group 2 with the whole bank system. Left: corporate portfolio; right: retail portfolio. Adverse macroeconomic conditions affect the corporate portfolio with a delay. Group 2 banks react to corporate events with less agility.

A preliminary survey of the IRD curves (cf., Figure 1) shows that the IRD can be useful to compare the riskiness of portfolios.

- The corporate portfolio reacts to the economic crisis of 2009–2010 with a delay in both groups.
- The 2016 system-wide asset quality review caused all banks to clean up their portfolios beforehand, as Group 1 banks have shown more agility and organization in this process. In comparison, for Group 2, the effect of this process is visible for as many as four quarters afterward. This contributes to the observed autoregressive property of the Group 2 corporate series, as discussed below.
- Group 2 exhibits a steep increase in IRD in 2013, which is most likely a reflection of the process of closure of CCB, one of the larger banks in the group.
- A greater volatility in the retail IRD is also confirmed by Table 1. This confirms the expectations that corporate clients are more carefully selected with greater individual attention from the banks than personal loans.

Table 2 furthermore reveals a difference between the two groups in the response of their corporate IRD to macroeconomic changes. In Group 1, the IRD exhibits a strong dependence on four-quarter-old dynamics of the GDP, while the portfolio in Group 2 reacts to changes in GDP much quicker—after only one quarter. A likely explanation for this difference is in the quality of clients the two types of banks attract: the banks in Group 1 can offer better conditions to larger and to less risky corporate clients, hence they are likely to react with a delay to adverse changes in economic conditions. A second possibility is that the larger banks in Group 1 have adapted much faster and better their financial reporting to the new accounting standard, hence the IRD has a different pattern of reflecting the rates of default. (This explanation is less likely because the difference is not confirmed in the retail portfolio.)

The result for Group 1 is confirmed with the data for the whole bank system as well. This is due to the fact that currently Group 1 is responsible for roughly 75% percent of corporate banking in the country.

The fact that the retail portfolios react immediately to changes in the unemployment rate is most likely explicable by the uncollateralized personal loans.

The major difference between the ARMA models for corporate portfolios (cf., Equations (9) and (10)) of the two groups is the presence of a negative autoregressive component for Group 2. This has the effect of a partial reversal of the past quarter’s (over) reaction. This is typical behavior of market followers. The strong positive moving-average component for all banks is evidence of regular management of the corporate portfolios in small and large banks in the country.

Likewise, the ARMA models for the retail portfolios (cf., Equation (11)) show a strong negative autoregressive component for all groups. This evidence confirms the understanding that the larger retail portfolios are managed collectively with a great degree of automation. In addition, the alternative retail model (12) for Group 2 shows a strong positive momentum from one quarter to the next. As historic data become more ample in the future, the dynamic of IRD for Group 2 could be a subject of more intricate modeling.

## 7. Conclusions

We define the *inferred rate of default* to approximate the rate of default using available systemic data. The resulting series exhibits the following similarities with the known properties of the rates of default:

1. The IRD reflects the rate of transition of the exposure of the portfolio from a regular state to the state of implied impairment. For this reason, it is a measure of the credit riskiness of the exposure.
2. The IRD similarly depends on macroeconomic factors such as RD. The IRD for corporate loans is explained by the growth in the GDP, and for retail, by the growth in the unemployment rate.
3. The IRD can be modeled using an asymptotic single risk factor, similar to the Vasicek model, and can be forecast similarly. While this type of model offers a slight advantage over less complex time-series models, they are found by banks to be less intuitive and their results are harder to manage.
4. The comparative analysis of IRD in different portfolios and bank groups is revealing of differences that are inherent in the types and quality of the client, as well as the style of management of the institutions.

The usefulness of the inferred rate of default to practitioners has two aspects. Firstly, the IRD can be applied for the measurement and benchmarking of the asset quality of banks’ credit portfolios. While a bank’s risk officer knows the actual rate of default of the portfolios under their management, they would have access to data from their peers and competitors only after the publication of the annual reports. Using IRD instead would allow comparison in a timely fashion. Secondly, and perhaps more importantly, it can be instrumental in computing replacements for missing values in a bank’s default rate distribution. In the process of building their models for credit loss analysis, small banks often find that their RD time series contain missing or defective data due to errors, other operational risks, or corporate events. In such cases, the comparison between the IRD and RD distributions could help with the correction of the data.

## Figures and Tables

**Table 1:** Summary statistics of the inferred rate of default by segment and bank group. Two segments, *corporate* and *retail*, and three portfolios, the total bank system, Group 1, and Group 2, are reported.

		Obs	Mean	Std. Dev.	Min	Max
**Bank System**	*Corporate*	48	0.0392	0.0360	0.0061	0.1253
	*Retail*	48	0.0296	0.0225	0.0073	0.0922
**Group 1**	*Corporate*	48	0.0421	0.0426	0.0050	0.1644
	*Retail*	48	0.0289	0.0234	0.0077	0.0940
**Group 2**	*Corporate*	48	0.0432	0.0355	0.0026	0.1185
	*Retail*	48	0.0393	0.0315	0.0063	0.0997

**Table 2:** Comparison between OLS regression with different lags (8). The coefficient α is not reported. The $R^{2}$ and β coefficients are reported along with the *p* values of their individual *t*-tests of significance. Corporate portfolios in Group 1 and the whole bank system show strong negative dependence of GDP at a 4-quarter lag. As the lag is reduced, the significance and the explanatory power of the model diminishes. Respectively, for corporate in Group 2, the best model is with lag 0. In the retail segments, all portfolios exhibit a strong positive relationship with the current unemployment rate. Note that the reversal of the relationship with stale data may be evidence of compensating for an over-reaction.

		Corporate			Retail		
**Lag**		**Bank System**	**Group 1**	**Group 2**	**Bank System**	**Group 1**	**Group 2**
τ=0	Coef.	−2.24	0.52	−7.82	1.68	1.39	1.71
	*p*-value	(0.442)	(0.898)	(0.008)	(0.000)	(0.020)	(0.009)
	$R^{2}$	0.01	0.00	0.16	0.50	0.13	0.16
τ=1	Coef.	−4.35	−3.66	−7.33	1.15	1.06	0.79
	*p*-value	(0.137)	(0.37)	(0.014)	(0.001)	(0.075)	(0.240)
	$R^{2}$	0.05	0.02	0.14	0.27	0.08	0.04
τ=2	Coef.	−6.68	−8.63	−5.65	0.61	0.60	0.41
	*p*-value	(0.021)	(0.033)	(0.058)	(0.079)	(0.315)	(0.547)
	$R^{2}$	0.13	0.11	0.09	0.08	0.03	0.01
τ=3	Coef.	−8.13	−12.05	−4.25	−0.22	−0.26	−0.39
	*p*-value	(0.004)	(0.002)	(0.136)	(0.536)	(0.670)	(0.577)
	$R^{2}$	0.19	0.21	0.06	0.01	0.01	0.01
τ=4	Coef.	−9.10	−14.40	−2.78	−1.14	−0.87	−1.49
	*p*-value	(0.001)	(0)	(0.313)	(0.000)	(0.147)	(0.028)
	$R^{2}$	0.25	0.31	0.03	0.32	0.06	0.13

**Table 3:** ARMA regressions for the *corporate* IRD. The models for the whole bank system and Group 1 are according to specification (9), and for Group 2, according to specification (10).

	Lag		β	ρ	θ	$\chi^{2}$
**Bank System**	τ=4	Coef.	−7.84	—	0.73	49.38
		*p*-value	(0.013)		(0.0001)	(0.0001)
**Group 1**	τ=4	Coef.	−13.57	—	0.83	69.05
		*p*-value	(0.035)		(0.0001)	(0.0001)
**Group 2**	τ=0	Coef.	−5.89	−0.52	0.87	49.92
		*p*-value	(0.029)	(0.023)	(0.0001)	(0.0001)

**Table 4:** ARMA regressions for the *retail* IRD. The models for all portfolios are according to specification (11). For Group 2, we report an alternative specification (12) as well.

		β	$\rho_{1}$	$\rho_{4}$	$\chi^{2}$
**Bank System**	Coef.	1.72	—	−0.31	43.46
	*p*-value	(0.001)		(0.031)	(0.0001)
**Group 1**	Coef.	1.29	—	−0.42	16.42
	*p*-value	(0.043)		(0.003)	0.0003
**Group 2**	Coef.	1.73	—	−0.44	20.38
	*p*-value	(0.023)		(0.0001)	(0.0001)
**Group 2 (Alt).**	Coef.	2.02	0.44	—	10.78
	*p*-value	(0.045)	(0.006)		(0.004)

**Table 5:** ARMA regressions for the *Z*-score of the IRD. The models for the whole banks system and Group 1 are according to specification (9), and for Group 2, according to specification (10). The results reveal that *Z*-scores positively correlate to GDP, as expected.

		β	ρ	θ	$\chi^{2}$
**Bank System**	Coef.	8.47		0.77	45.01
	*p*-value	(0.015)		(0.0001)	(0.0001)
**Group 1**	Coef.	14.57		0.85	90.25
	*p*-value	(0.039)		(0.0001)	(0.0001)
**Group 2**	Coef.	5.62	−0.45	0.86	46.13
	*p*-value	(0.068)	(0.048)	(0.0001)	(0.0001)

**Table 6:** ARMA regressions for the *Z*-score of the retail IRD. The models for all portfolios are according to specification (11). The results reveal that *Z*-scores for the retail portfolio negatively correlate with the unemployment rate, as expected.

		β	$\rho_{4}$	$\chi^{2}$
Bank System	Coef.	−2.47	−0.38	56.23
	*p*-value	(0.0001)	(0.018)	(0.0001)
Group 1	Coef.	−1.92	−0.43	17.96
	*p*-value	(0.023)	(0.002)	(0.0001)
Group 2	Coef.	−1.88	−0.45	21.40
	*p*-value	(0.011)	(0.0001)	(0.0001)

**Table 7:** Comparison between the accuracy of the two models. Row 1 and row 2 present the standard error of the log-ARMA time-series analysis, and ASRF estimation, respectively. The standard quadratic deviation is computed between the RDI and the estimated PI: $Std.Err.={\left(\sum_{t}{\left(RDI_{t}-PI_{t}\right)}^{2}\right)}^{1/2}$.

	Corporate			Retail		
	**Bank System**	**Group 1**	**Group 2**	**Bank System**	**Group 1**	**Group 2**
Log-ARMA	0.0184	0.0245	0.0865	0.0109	0.0180	0.0254
ASRF	0.0180	0.0242	0.0841	0.0104	0.0176	0.0248

## Data Availability

Publicly available datasets were analyzed in this study. The data for the bank groups asset quality can be found at the website of the Bulgarian National Bank: https://www.bnb.bg/BankSupervision/index.htm (accessed on 7 March 2022) and the macroeconomic indicators are available from the National Statistical Institute: https://www.nsi.bg/en (accessed on 8 March 2022).

## Abbreviations

The following abbreviations are used in this manuscript:

ECL	Expected credit loss
IFRS 9	The International Financial Reporting Standard 9
IRD	Inferred rate of default
PD	Probability of default
PII	Probability of inferred impairment
PIT	Point-in-time
RD	Rate of default
TTC	Through-the-cycle
